# Aging on the road: neural and behavioral correlates of cognitive control during distracted driving

**DOI:** 10.3389/fnagi.2026.1864407

**Published:** 2026-09-15

**Authors:** Emad Alyan, Stephan Getzmann, Edmund Wascher, Melanie Karthaus

**Affiliations:** Department of Ergonomics, Leibniz Research Centre for Working Environment and Human Factors, Dortmund, Germany

**Keywords:** aging, cognitive control, driving, EEG, eye blinks, MVPA

## Abstract

**Objective:**

Aging increases crash risk through failures to prioritize safety-critical actions amid competing information. The neural basis of adaptive cognitive control under these demands remains insufficiently understood.

**Methods:**

This study examined age effects on braking prioritization in 44 drivers (23 younger, 21 older) using a car-following simulation with lane keeping, in which participants withheld sign responses whenever brake signals took priority. Participants completed Sign (button press to classify road signs), Brake (pedal press to the brake lights of the car ahead), and dual-demand signBrake trials (simultaneous Sign and Brake, braking prioritized). Measures included reaction time, errors, mean absolute steering-wheel angle, blink metrics, ERPs (N1, P3, and LFN), time-frequency power, and multivariate pattern analysis (MVPA) of age decoding.

**Results:**

Older adults exhibited selective signBrake costs, with slower and less accurate braking than younger adults, and a larger mean absolute steering-wheel angle. In both groups, steering-wheel angle decreased from the pre- to the post-stimulus period, with the largest drop in signBrake. Blink rate did not differ by age, but younger adults exhibited longer blink durations and larger amplitude-velocity ratios, suggesting differences in how blinks punctuate attentional sampling during driving. The LFN (late frontal negativity), a preparatory index of response control, showed weaker condition-related differentiation in older adults, who also exhibited larger N1 but smaller P3 amplitudes. This pattern fits greater early visual attentional selection and weaker later evaluation and context updating. Time-frequency analyses revealed reduced frontal theta, parietal alpha, and central beta power in older adults across conditions. Central beta demonstrated the strongest Age × Condition modulation, driven mainly by older adults, while younger adults showed no reliable condition differences, which fits weaker post-stimulus attentional gating and altered sensorimotor tuning during action selection. MVPA decoded age group above chance after stimulus onset in all three conditions. Cross-condition decoding succeeded across train-test pairings, consistent with a distributed age-related neural signature that generalizes across contexts.

**Conclusion:**

These findings tie signBrake performance costs to weaker preparatory control differentiation and altered post-stimulus dynamics that support braking under interference. This reduced flexibility may contribute to age-related declines in driving safety in moments that call for rapid, goal-directed control.

## 1 Introduction

The aging of the driver population raises important public safety concerns. Older drivers show some of the highest automobile crash and fatality rates per mile driven of any age group (Tefft, 2017). This pattern cannot be explained simply by greater exposure on the road. Age-related declines in sensory functions, including visual acuity, contrast sensitivity, and processing speed, occur alongside changes in motor control and higher-order cognition. These shifts undermine the complex skill set needed for safe driving (Carr and Babulal, 2023; Ortiz-Peregrina et al., 2020). They slow reaction time and raise the probability of driving errors (Doroudgar et al., 2017). This can destabilize vehicle control. Drivers then make more variable steering adjustments and keep lanes less consistently than younger drivers (Ortiz-Peregrina et al., 2020). Driving is attentionally distributed where drivers must continuously monitor road signs, mirrors, in-car displays, and surrounding traffic, often under time pressure (Michaels et al., 2017). Dual-task and distraction studies consistently show that handling visual or cognitive tasks at the same time leads to longer brake reaction times, more errors, and lane-keeping difficulties (Karthaus et al., 2018). Safety-critical brake-light signals must be prioritized over less urgent information, for example road signs or displays (Huizeling et al., 2020).

From a cognitive control viewpoint, safe driving entails both proactive control and selective inhibition. Proactive control represents a continuous and goal-directed process characterized by holding relevant information for anticipation and interference prevention (Mäki-Marttunen et al., 2019). This process helps with attentional and motor priming for probable upcoming events (Grisetto et al., 2023; Wascher et al., 2018). Meanwhile, reactive control entails a temporary control process activated after conflict and unexpected stimuli for identification and resolution (Grisetto et al., 2023). Safety-critical braking is a clear example of reactive inhibition and often requires canceling or withholding prepotent responses, an inhibitory function that is essential for adaptive behavior (Aron, 2011). Older adults frequently show reduced efficiency of proactive control and a greater reliance on reactive strategies, particularly under multitasking or high-load conditions (Czernochowski et al., 2010). However, this shift varies across older adults. Some older drivers maintain effective proactive control when task structure or motivation supports it (Xiang et al., 2016). Age-related difficulties in refocusing attention and suppressing task-irrelevant signals have been highlighted across everyday activities, including car driving in complex traffic situations (Getzmann et al., 2023). Older adults show reduced resistance to distractor interference and less efficient inhibition of prepotent responses. Thus, situations that require withholding sign-related actions, then rapid braking, pose a theoretically important challenge in later life. Age also affects eye movement control, especially blinking, which shows the relationship between visual attention, alertness, and muscle coordination. Older adults often blink more frequently but for shorter periods. This aligns with their need to stay visually alert (Alyan et al., 2025; Caffier et al., 2003). Additionally, kinematic measures, including the peak amplitude-velocity ratio, reflect eyelid movement control (Kleifges et al., 2017). The effects of age on these kinematic measures are still poorly understood.

These control processes are recorded by electroencephalography (EEG) at millisecond resolution. It provides first-hand information on anticipation, attention and decision dynamics (Getzmann et al., 2018). The late frontal negativity (LFN) is a prolonged frontal negative deflection linked with cognitive flexibility and solving of the opposition of response needs (Kononowicz and Penney, 2016). It is seen most strongly when there is a need to concurrently balance competing response mappings and has been viewed as a neural signal of preparatory control over action priorities (Dirnberger et al., 2010; Kononowicz and Penney, 2016; Wild-Wall et al., 2007). Such competing demands might become less flexible in older adults as evidenced by changes in LFN amplitude and topography of the scalp (Kropotov et al., 2016). Early sensory event-related potentials (ERPs) include the occipital N1, which tracks attentional gain control and sensory prioritization. Older adults typically show larger N1 amplitudes in demanding or safety-relevant settings, fitting compensatory upregulation of visual attention (Alyan et al., 2025; Finnigan et al., 2011). The centro-parietal P3 reflects stimulus evaluation and context updating. With age, P3 amplitude often drops and latency lengthens, a common marker of slower processing (Alyan et al., 2025; Fields, 2023; Shubhadarshan and Gaiwale, 2024). These ERP measures show how aging shapes anticipation, attention, and evaluation in complex tasks like driving.

Time-frequency spectral analyses complement ERP measures and add a second view of EEG activity. They capture non-phase-locked oscillations that support large-scale cognitive control. For example, frontal theta marks cognitive control and conflict monitoring. Older adults often show lower theta power. Yet higher frontal theta can signal compensatory engagement that supports performance (Springer et al., 2023; Tafuro et al., 2019). Alpha-band activity mediates attentional gating and inhibition of irrelevant information. Older adults often show reduced alpha power and weaker synchronization, pointing to weakened or reconfigured top-down control (ElShafei et al., 2020). Beta-band oscillations over sensorimotor regions reflect motor preparation and execution. Aging alters beta desynchronization and cortico-muscular coupling, consistent with adaptive motor recalibration (Van Hoornweder et al., 2022). Across the theta, alpha, and beta bands, these shifts indicate compensatory neural reorganization that may help preserve attention and control, even with relatively stable overt behavior.

ERP and spectral measures clearly show age-related changes in attention and control, but they provide only a limited view of how information is distributed across neural networks. Traditional univariate ERP and time-frequency power analyses may therefore miss subtle representational differences between age groups. Multivariate pattern analysis (MVPA) provides a well-suited framework for decoding distributed EEG and MEG activity (Alyan et al., 2023; Cichy and Pantazis, 2017). It captures how neural representations evolve over time. Recent research indicates that aging alters the temporal precision and distinctiveness of neural representations (Wan et al., 2024). This pattern is consistent with reduced stability or compensatory reorganization of brain activity. Yet MVPA has been applied rarely to ecologically valid tasks, such as braking behavior with competing signs and brake signals. These tasks require resolving both signals. The representational basis of age-related differences in such scenarios remains largely unknown.

In the present study, younger and older adults completed a simulated driving task with responses to road signs, brake-light events, or dual-demand trials that presented both signals and required prioritizing braking. Measures included behavior (reaction time, accuracy), steering control, ERPs (N1, P3, and LFN), time-frequency power (theta, alpha, beta), and MVPA-based decoding of task and age information. The primary goal was to characterize how aging affects the ability to prioritize brake-light responses when competing sign information is present, and to link any resulting performance costs to changes in preparatory, sensory, and decision-related EEG markers. The central prediction was that dual-demand (signBrake) trials would impair performance relative to single-signal Sign and Brake trials, with higher costs in older than younger adults, accompanied by a larger steering-wheel angle and differing blink patterns by age group. Older adults were also expected to show less flexible LFN-related preparation and age-related changes in sensory (N1) and evaluative (P3) ERP components, consistent with altered selective attention and control in aging, along with age-dependent changes in the theta, alpha, and beta frequency ranges across conditions reflecting differences in control over physical and sensory actions. Finally, time-resolved MVPA of time-frequency power was expected to show above-chance age classification within each condition after stimulus onset, with cross-condition generalization consistent with a shared neural signature of aging. The central hypothesis behind this study is that aging primarily impairs interference-related action selection, that is, choosing the contextually correct response when two competing response tendencies are cued simultaneously, rather than reactive braking itself or general response speed. These predictions were tested with confirmatory analyses. In contrast, the pre- versus post-stimulus steering contrast and its interaction with condition, the whole-scalp cluster-based permutation maps, the decoding-behavior correlation, and all post-hoc pairwise contrasts should be considered exploratory.

## 2 Materials and methods

### 2.1 Participants

Forty-four licensed drivers participated in the experiment. The sample included 23 younger adults (ages 19 to 25 years, *M* = 22.8, *SD* = 2.2) and 21 older adults (ages 65 to 74 years, *M* = 68.6, *SD* = 2.4). The overall sample comprised 22 men and 22 women. Within the younger group, 11 were male and 12 female participants, while the older group included 11 male and 10 female participants. A *post hoc* sensitivity analysis (G*Power 3.1; α = 0.05, 1 - β = 0.80) on this predetermined sample size indicated that this sample size (*N* = 44), given a 2 × 3 mixed design, detects Cohen’s *f* ≥ 0.19 for within-subject and Age × Condition effects, *f* ≥ 0.35 for between-subject Age main effects, and *d* ≥ 0.87 for Welch’s two-sample *t*-tests. All participants reported normal or corrected-to-normal vision and held a valid driving license. They provided written informed consent prior to participation and received €40 as monetary compensation. The study was conducted in a driving simulator at the Leibniz Research Centre for Working Environment and Human Factors (IfADo) in Dortmund and received approval from the local ethics committee. Participants had normal or corrected-to-normal hearing, no current or past neurological disorder, and no use of medication affecting the central nervous system. Driving history and routines were assessed by questionnaire. Annual mileage did not differ significantly between age groups (young, *M* = 9,400 km, *SD* = 7,543; old, *M* = 11,381 km, *SD* = 4,994), *t* (42) = −1.02, *p* = 0.315, and all participants drove at least twice a week. Participation required that individuals had not previously taken part in another simulator experiment within this project. Participant characteristics are summarized in Table 1.

**Table 1:** Behavioral performance and participant characteristics by age group.

Measure	Younger (*n* = 23)	Older (*n* = 21)	Age contrast
Age (years)	22.8 (2.2)	68.6 (2.4)	-
Sex (M/F)	11/12	11/10	-
Annual mileage (km)	9,400 (7,543)	11,381 (4,994)	*p* = 0.315
RT, Sign (ms)	889 (122)	934 (111)	*p* = 0.201, p_fdr_ = 0.201
RT, Brake (ms)	575 (92)	651 (134)	*p* = 0.037, p_fdr_ = 0.054
RT, signBrake (ms)	605 (98)	776 (149)	*p* < 0.001, p_fdr_ < 0.001
Error rate, Sign (%)	7.00 (6.45)	5.23 (3.12)	*p* = 0.250, p_fdr_ = 0.356
Error rate, Brake (%)	1.23 (1.93)	1.83 (2.30)	*p* = 0.356, p_fdr_ = 0.356
Error rate, signBrake (%)	3.04 (3.34)	11.67 (6.97)	*p* < 0.001, p_fdr_ < 0.001
Interference cost, RT (ms)	30	125	*p* < 0.001
Interference cost, errors (%)	1.8	9.8	*p* < 0.001

Values are *M (SD)*. Interference cost is signBrake minus Brake. RT = response time; p_fdr_ = FDR-corrected *p*-value.

### 2.2 Task and procedure

Participants performed a continuous car-following task in a fixed-base driving simulator equipped with a three-screen panoramic display (three 60-inch screens), a standard steering wheel, and functional pedals, without a motion platform. They followed a lead vehicle at a fixed distance of about 15 m. The lead vehicle traveled at a constant speed of 50 km/h. The road was straight, with two lanes, through a low-stimulus grassland environment (Karthaus et al., 2021). Participants were instructed to keep their own vehicle as precisely as possible in the right lane. They also counter-steered against a lateral crosswind whose timing was independent of the critical events. The crosswind pushed the car to the left or right. Critical events were brake-light illuminations of the lead vehicle and brief location signs. The signs showed either German city names or country names. Both event types lasted 500 ms and occurred at pseudo-random intervals of 6–8 s. Brake lights served as safety-relevant signals. Each time they flashed, participants had to press the brake pedal as quickly as possible. Location signs appeared on roadside panels. They required participants to press a left- or right-hand button on the steering wheel according to an individually assigned mapping (city vs. country name), with the mapping counterbalanced across participants. Drivers were explicitly instructed that the driving task (steering and braking) always had priority over sign responses. Three trial types were analyzed. In the Brake condition, only the brake lights of the lead vehicle appeared and required a rapid braking response. In the Sign condition, only a location sign was presented and required the corresponding button press. In the combined signBrake condition, brake lights and a location sign occurred at the same time. The sign remained visible throughout, and participants were instructed to brake and withhold only the sign-related button press, so signBrake required a single motor response under response competition. Brake-light events served as the primary safety signal, while isolated signs served as secondary task stimuli. Responses within 100–2,500 ms of stimulus onset counted as valid; later ones, up to the next critical event (6–8 s), were scored as omissions. Incorrect and omitted trials were dropped from the RT analyses along with their RTs.

The data come from a within-subject intervention study (Karthaus et al., 2021). Participants attended three sessions roughly two weeks apart. Each session included a PRE measurement (about 34 min, split into two 17-min blocks), one of three intervention measurements (acoustic cue, acoustic feedback, or a no-intervention control, each about 34 min and split into two blocks), and a POST measurement (about 34 min, split into two blocks). Intervention order was counterbalanced across sessions and participants. Because each of the three intervention types occurred in exactly one session, only one session per participant carried the no-intervention CONTROL measurement, while every session also contributed its own PRE and POST measurement. Only measurements without active cue or feedback were used here (three PRE, one CONTROL, three POST per participant) with 96 Brake-light, 96 Sign, and 96 signBrake events per measurement. Continuous EEG recordings from these measurements were concatenated per participant before epoching. Session position was not entered as a factor in any analysis (Figure 1).

**Figure 1:**
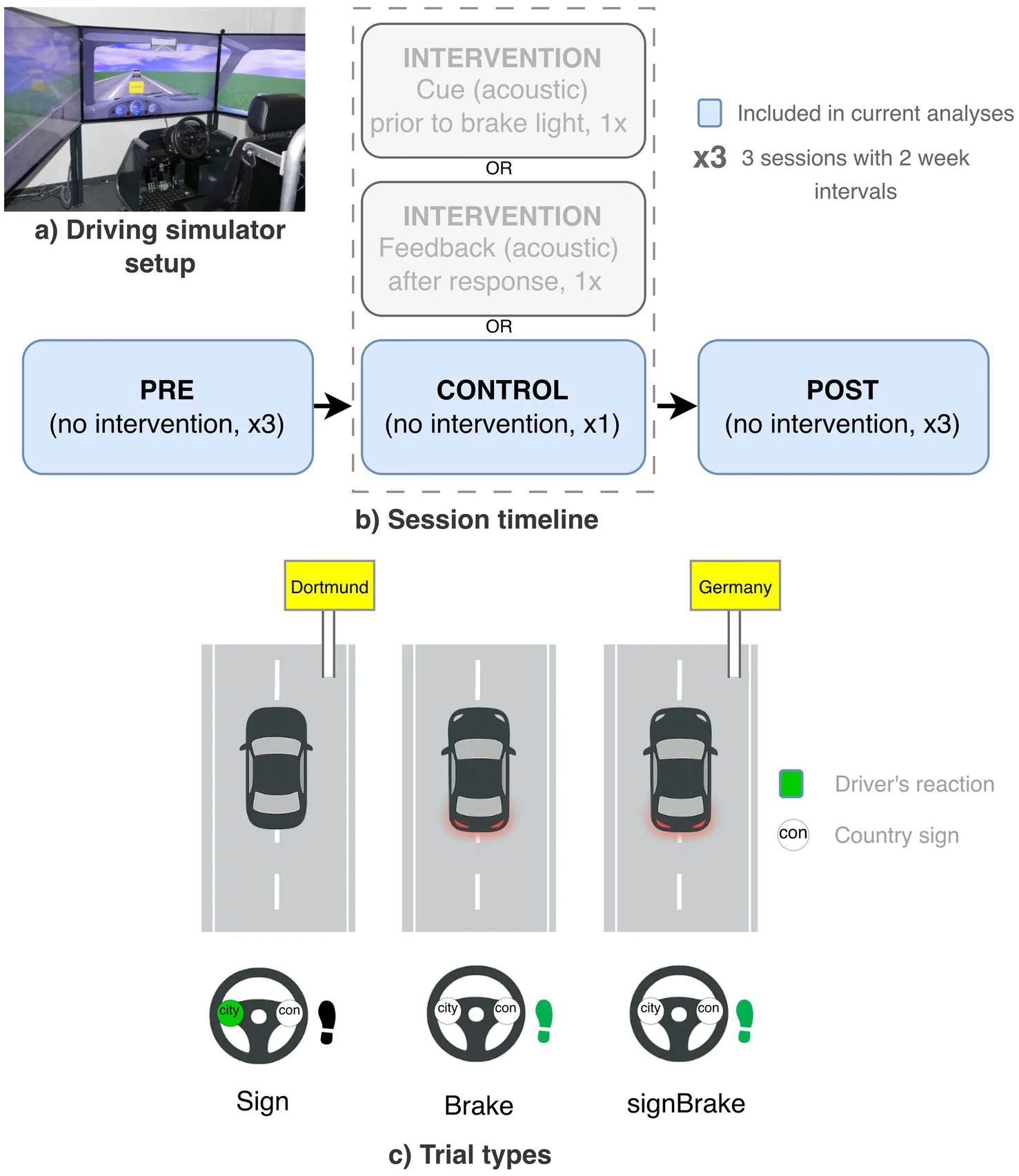
Experimental design and trial structure. **(a)** driving simulator setup. Participants completed the task in a fixed-base driving simulator equipped with a three-screen panoramic display, a standard steering wheel, and functional pedals. **(b)** Sessions were separated by 2-week intervals. The gray dashed box indicates the intervention phase, in which each participant was assigned one of three conditions: Acoustic cue, acoustic feedback, or no-intervention control. Analyses used the no-intervention measurements only: three PRE and three POST sessions (×3 each), plus one CONTROL session (×1). **(c)** Trial types. Schematic road scenes depict three conditions: in Sign, participants responded to a city/country sign with a button placed on the steering-wheel (green button); in Brake, they reacted to illuminated brake lights with a foot press on the pedal (green foot); and in signBrake, simultaneous onset of brake lights and a city/country sign required a braking response while inhibiting the response to the sign.

### 2.3 Data acquisition

Participants performed a combined lane-keeping and braking task in a fixed-base driving simulator (ST Sim, ST Software B. V., Groningen, The Netherlands). They followed a lead vehicle at constant speed on a straight two-lane road. The simulator continuously recorded vehicle dynamics and pedal activity. This yielded time-stamped braking events. These events were later synchronized with the EEG recordings. Continuous EEG was acquired with a BioSemi amplifier (ActiveTwo; BioSemi; Amsterdam, The Netherlands). Thirty-two Ag/AgCl scalp electrodes were used, arranged according to the 10–20 system and including two mastoid electrodes. Signals were sampled at 2048 Hz. All channels were recorded concurrently. The data were stored for later offline preprocessing and analysis.

### 2.4 EEG preprocessing

EEG preprocessing was run in MATLAB (R2025b, MathWorks, Natick, MA) using EEGLAB v2025.0.0 (Delorme and Makeig, 2004) and custom scripts. Raw EEG data were band-pass filtered from 0.1 to 40 Hz with a Hamming-windowed FIR filter. The data were then downsampled to 256 Hz. This kept the dataset smaller while preserving the frequencies of interest. Narrowband nuisances, line noise and its harmonics, were addressed with Zapline Plus (Klug and Kloosterman, 2022). Noisy channels were then detected with clean_rawdata (correlation threshold = 0.8 to a robust reference, maximum broken-time fraction = 0.3, ten detection iterations, detection-frequency threshold = 0.5). Channels that consistently failed these criteria were marked as bad. They were interpolated using spherical splines. The data were then re-referenced to the common average.

Independent component analysis (ICA) was performed on the average-referenced data. A 1.5 Hz high-pass filter (zero-phase FIR, order = 1,650) was applied first. This choice aimed to stabilize ICA in naturalistic EEG (Frølich and Dowding, 2018). The data were then decomposed into statistically independent components using Adaptive Mixture ICA (AMICA) (Palmer et al., 2012), with a single model, automatic sample rejection (10 rejection iterations, 3-*SD* threshold), a maximum of 2000 iterations, and four parallel threads. The resulting spatial filters were back-projected onto the original average-referenced EEG. Components were automatically labeled with the ICLabel algorithm (Pion-Tonachini et al., 2019). Only components dominated by cortical activity were retained. Components were excluded if brain probability was below 30%. They were also excluded if any non-neural class exceeded 30% probability. These classes included eye movements, muscle or cardiac activity, line noise, channel noise, and other sources. Finally, EEG data were epoched from −2000 to +2000 ms around the stimulus. The data were cleaned with EEGLAB pop_autorej, which first removes epochs with extreme excursions (any sample beyond ±1,000 μV) and then iteratively rejects statistically improbable epochs exceeding a 5 *SD* joint-activity criterion (maximum 5% removed per iteration). Epochs from trials with incorrect behavioral responses were also removed. On average, 487 ± 91 Sign epochs, 512 ± 91 Brake epochs, and 483 ± 97 signBrake epochs were retained per participant.

### 2.5 Data analysis

#### 2.5.1 Behavioral and steering measures

For each condition, the numbers of correct and incorrect trials were used to compute error rates. Error rates were defined as the percentage of incorrect responses relative to all valid trials. A trial was scored as an error when the response did not match the condition rule: in Sign (correct response = correct-side left/right button press, no brake), errors were a wrong-side button press, any brake-pedal press (with or without a button press), or no response within the response window; in Brake (correct response = brake-pedal press only), errors were no brake-pedal press within the window (with or without an extraneous button press) or a button press accompanying the brake; in signBrake (correct response = brake-pedal press only), errors were any left/right button press (with or without brake), no brake-pedal press within the window, or no response at all. Mean response times (RTs) were calculated for each participant and condition. RTs were averaged across correct trials only. In Sign, RT was the latency of the correct left/right button press relative to sign onset; in Brake and signBrake, RT was exclusively the brake-pedal-press latency, taken relative to the corresponding stimulus event (brake-light onset in Brake, and the simultaneous sign/brake-light onset in signBrake). These subject-level error and RT indices served as the behavioral input for all group-level analyses. Driving-related variables, such as steering-wheel angle, were extracted from the synchronized simulator data. They were time-locked to the same events used for EEG epoching. The absolute steering-wheel angle was averaged within pre-stimulus (−3,000 to 0 ms) and post-stimulus (0 to +3,000 ms) windows, with larger values indicating greater steering-wheel displacement. This was done relative to stimulus onset, separately for each condition and participant.

#### 2.5.2 Eye-blink metrics

Blink properties were derived from the BLINKER outputs associated with the blink-related IC (Alyan et al., 2025; Kleifges et al., 2017). For each participant, blink events were aligned with the EEG event list via their sample indices. This made it possible to assign individual blinks to the three task conditions. Blink rate per minute was calculated as the number of blinks divided by the effective recording time within each condition. Additional blink descriptors were also extracted. These included positive and negative amplitude-velocity ratios (pAVR, nAVR), blink duration, and inferred closing and reopening times. They were averaged across blinks within each participant. Trials from the three tasks were randomly intermixed. For that reason, blink measures were collapsed across conditions. This yielded a single estimate per participant.

#### 2.5.3 Event-related potentials

Event-related potential analyses were based on the condition-specific EEG datasets. For each subject and condition, continuous data were segmented into epochs from −300 to +1,000 ms relative to stimulus onset. The data were baseline-corrected using −200 to 0 ms. Single trials were then averaged. This produced one ERP waveform per channel. To reduce dimensionality and match clear *a priori* hypotheses, three regions of interest (ROIs) were defined. These were frontal (F3, Fz, F4, FC3, FCz, FC4), parietal (P3, Pz, P4), and occipital (Oz, O1, O2, PO3, POz, PO4). Within each ROI, regional ERPs were computed by averaging across the listed electrodes. This yielded one summary waveform per condition and subject. ERP components were characterized using mean amplitudes in pre-specified latency windows. The components quantified were an LFN over frontal sites in the late post-stimulus interval (300–500 ms), an early visual N1 over occipital sites (80–250 ms), and a centro-parietal P3 over parietal sites (300–400 ms). Component quantification was based on grand-average waveforms computed separately for each condition (collapsed across age groups). Within each component-specific latency window, the maximal deflection of the expected polarity (P3 positive; LFN and N1 negative) was located and treated as the grand-average peak latency. For each subject, condition, and component, the mean voltage within a 20-ms window (±10 ms) centered on this latency was extracted as the ERP amplitude. These amplitudes were entered into the mixed-effects ANOVAs described in the Statistical analysis section.

#### 2.5.4 Time-frequency analysis

Time-frequency analyses were conducted on the same correct-response epochs used for the ERP analyses. For each participant and condition, continuous cleaned data were epoched from −2000 to +2000 ms relative to the stimulus event. Time-frequency decomposition was then performed for every channel using EEGLAB’s pop_newtimef routine. Complex Morlet wavelets with variable cycle lengths (cycles = [3 0.8]) were applied with a window size of 256 samples and a padding ratio of 1. For each channel and condition, event-related spectral perturbations (ERSPs) were computed across trials. ERSP values were baseline-corrected in decibels relative to a pre-stimulus window from −500 to −300 ms. The resulting ERSP maps comprised frequency by time matrices per subject, condition, and channel. To focus on the frequency and time ranges relevant for cognitive control and braking behavior, the whole-scalp analyses were restricted to 4–30 Hz and to the interval from −400 to +1,400 ms relative to stimulus onset. ERSPs were averaged across all scalp EEG channels and compared between age groups using non-parametric cluster-based permutation tests. The null distribution was generated by randomly reassigning participants to young and old and recomputing the young-old difference 20,000 times. Each permuted map was z-scored relative to the permutation-based mean and variance, and clusters of contiguous supra-threshold pixels (|z| ≥ 1.96, pixel-wise alpha = 0.025, two-sided) were quantified using a cluster-mass statistic. The 97.5th percentile of the permutation-based maximum cluster mass defined the cluster-level threshold (familywise alpha = 0.05). Only clusters exceeding this threshold were retained as significant in the final z-maps. As a follow-up secondary analysis, the average ERSP power was also computed for predefined ROIs, including frontal theta (5–7 Hz; F3, Fz, F4, FC3, FCz, and FC4), parietal alpha (8–13 Hz; P3, Pz, P4), and central beta (14–30 Hz; C3, Cz, C4, CP3, CPz, and CP4). This was done to test whether age-related differences were more apparent in oscillatory systems linked to cognitive control, posterior attentional and visual processing, and sensorimotor response regulation.

#### 2.5.5 Multivariate time-frequency decoding of age group

Does age leave a measurable fingerprint in the time-frequency data? Age-related information in the time-frequency domain was quantified with time-resolved multivariate decoding of the ERSP maps described above. For each participant, condition (Sign, Brake, signBrake), and EEG channel, single-trial ERSPs were averaged across trials. The maps were then cropped to 4 to 30 Hz and −600 to 1,500 ms relative to stimulus onset. For each condition, the time-frequency matrices were concatenated across frequencies and channels to form feature vectors. This yielded a tensor of size S × (*F* × C) × T, where S is the number of participants, *F* = 27 frequency bins (4–30 Hz), C = 30 channels, and T = 141 time points (−600 to +1,500 ms). The classifier was run independently at each time point. At each time point, age group (younger vs. older) was decoded using L2-regularized ridge logistic regression (lambda = 1) applied to z-scored features. Z-scoring was computed across training participants within each leave-one-subject-out (LOSO) iteration and subsequently applied to the held-out participant. Generalization performance was assessed with LOSO cross-validation, separately for within-condition decoding (training and testing on the same condition) and cross-condition decoding (training on condition A, testing on condition B). For every train-test pairing and time point, each participant yielded a single 0/1 correctness indicator (1 = correctly classified age group, 0 = misclassified). The decoding accuracy of time courses was then derived as the mean proportion correct across participants, with standard errors estimated across participants.

To determine whether decoding exceeded chance level (0.5), a one-dimensional cluster-mass sign-flip permutation test was applied to the subject-wise accuracy values. For each decoding curve, *t*-statistics indexing the difference between accuracy and chance across participants were thresholded at a two-sided pixelwise alpha = 0.05. Cluster masses (sum of |*t*| across contiguous suprathreshold bins; minimum length = 2 bins) were compared against a null distribution generated from 20,000 random sign flips, and clusters exceeding the 95th percentile of this distribution (cluster-level alpha = 0.05) were deemed significant. Finally, classifier weights from the ridge models were averaged in absolute value over frequencies and significant time bins and visualized as scalp topographies to index the relative importance of each channel.

### 2.6 Statistical analysis

All statistical analyses were run in MATLAB (R2025b), and each model matched the dependent measure. Subject-level summaries of behavior (mean response times, percent errors), steering (mean steering-wheel angle in pre- and post-stimulus windows), ERP amplitudes, and region-of-interest (ROI) power were analyzed with mixed-design ANOVAs. Models included a between-subjects factor Age group (younger, older) and a within-subjects factor Condition (Sign, Brake, signBrake). Sphericity was evaluated with Mauchly’s test, and Greenhouse–Geisser (GG) corrections were applied to all within-subjects analyses. For steering measures, Time (pre, post) was added as an additional within-subjects factor. Independent-samples age-group comparisons were conducted using two-tailed Welch’s *t*-tests, used throughout given unequal group sizes and heterogeneous variances across conditions (Delacre et al., 2017). Multiple comparisons were controlled within each family of three condition-wise contrasts per dependent measure using the Benjamini-Hochberg false discovery rate (FDR) procedure (Benjamini and Hochberg, 1995). Effect sizes are reported as Cohen’s *d*. Eye-blink parameters (blink rate, blink duration, amplitude-velocity ratios, and closing/reopening times), averaged across the three task conditions, were compared between age groups using Welch’s *t*-tests. Event-related spectral perturbations (4–30 Hz; −600 to 1,500 ms) were computed for each participant and condition. EEG frequency band powers in predefined ROIs (frontal theta, parietal alpha, and central beta; 350–1,000 ms) were submitted to mixed-design ANOVAs, and condition-wise age-group contrasts were further examined using Welch’s *t*-tests with FDR correction. Age-related differences in the full time-frequency maps were assessed with cluster-based permutation tests on independent-samples contrasts, employing a pixel-wise threshold of alpha = 0.025 and cluster-mass control of the familywise error rate at alpha = 0.05. Post-hoc pairwise comparisons following ANOVAs were adjusted using the Tukey–Kramer procedure as implemented in MATLAB’s multcompare function. Age-by-predictor interactions were subsequently tested in linear regression models including group, predictor, and their interaction as fixed effects. Finally, multivariate age decoding was performed using time-resolved ridge-regularized logistic regression on multichannel time-frequency power, and accuracy time courses were tested against chance (0.5) using one-dimensional cluster-based permutation tests.

## 3 Results

### 3.1 Behavioral performance

Error rates and response times (RTs) were analyzed with a 2 (Age group: younger, older) × 3 (Condition: Sign, Brake, signBrake) mixed-design repeated-measures ANOVA. Figure 2 summarizes condition-wise means and standard errors for each age group (see Table 1). In the Sign condition, both age groups showed relatively modest error rates (younger: *M* = 7.00%, *SD* = 6.45; older: *M* = 5.23%, *SD* = 3.12). Errors were rare in the Brake condition (younger: *M* = 1.23%, *SD* = 1.93; older: *M* = 1.83%, *SD* = 2.30). In contrast, the signBrake condition elicited more errors overall, particularly in older adults (younger: *M* = 3.04%, *SD* = 3.34; older: *M* = 11.67%, *SD* = 6.97). Collapsed across age groups, error rates differed significantly between conditions, *F*(2,84) = 32.58, *p* < 0.001, p_GG_ < 0.001, η^2^_p_ = 0.44, with the fewest errors in Brake and higher errors in the sign-related conditions. Older adults committed more errors overall than younger adults (younger: *M* = 3.76%, older: *M* = 6.24%), *F*(1,42) = 5.90, *p* = 0.020, η^2^_p_ = 0.12. The Age group × Condition interaction was significant, *F*(2,84) = 25.72, *p* < 0.001, p_GG_ < 0.001, η^2^_p_ = 0.38, indicating that age differences were contingent on task demands. Follow-up Welch’s *t*-tests confirmed that age differences in error rate were not significant in the Sign condition, *t*(32.40) = 1.17, *p* = 0.250, p_fdr_ = 0.356, *d* = 0.34, 95% CI [−1.3, 4.8], or in the Brake condition, *t*(39.20) = −0.93, *p* = 0.356, p_fdr_ = 0.356, *d* = −0.28, 95% CI [−1.9, 0.7]. However, in signBrake, older adults made substantially more errors than younger adults (11.67% vs. 3.04%), *t*(28.14) = −5.16, *p* < 0.001, p_fdr_ < 0.001, *d* = −1.60, 95% CI [−12.1, −5.2].

**Figure 2:**
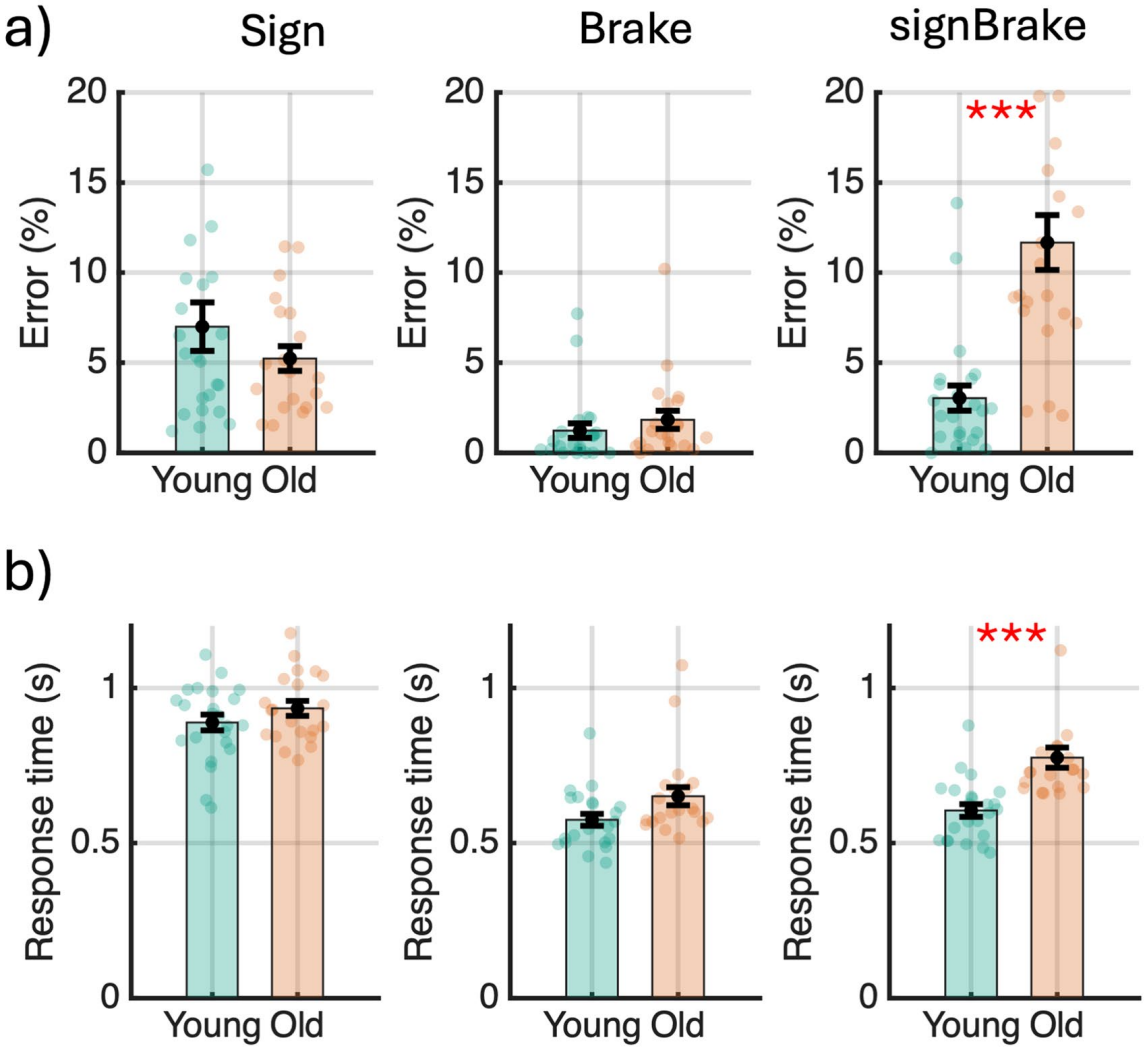
Behavioral performance by age group and condition. Panel **(a)** depicts error rates (%) and Panel **(b)** response times (RTs, s) for younger and older adults in the Sign, Brake, and signBrake conditions. Bars represent group means, dots mark individual participants, and error bars indicate the standard error of the mean. Significance stars denote age-group differences (Welch’s *t*-tests, FDR-corrected, *** *p*fdr < 0.001). Older adults showed higher error rates and RTs than younger adults only in signBrake, whereas age differences in Sign and Brake did not reach significance.

Response times showed a related, though not identical, pattern. In the Sign condition, younger adults responded with a mean RT of 889 ms (*SD* = 122), whereas older adults responded with 934 ms (*SD* = 111). In the Brake condition, responses were faster overall (younger: *M* = 575 ms, *SD* = 92; older: *M* = 651 ms, *SD* = 134). In the signBrake condition, younger adults responded on average in 605 ms (*SD* = 98) and older adults in 776 ms (*SD* = 149). Across age groups, RTs differed markedly between conditions, *F*(2,84) = 222.40, *p* < 0.001, p_GG_ < 0.001, η^2^_p_ = 0.84, with the shortest RTs in Brake, intermediate RTs in signBrake, and the longest RTs in the Sign condition. Older adults responded more slowly overall than younger adults (younger: *M* = 689 ms, older: *M* = 787 ms), *F*(1,42) = 9.55, *p* = 0.004, p_GG_ = 0.004, η^2^_p_ = 0.19. The Age group × Condition interaction was also significant, *F*(2,84) = 9.86, *p* < 0.001, p_GG_ = 0.002, η^2^_p_ = 0.19. Pairwise age-group comparisons indicated no significant age difference in RTs in the Sign condition, *t*(42.00) = −1.30, *p* = 0.201, p_fdr_ = 0.201, *d* = −0.39, 95% CI [−116.4, 25.2]. By contrast, older adults were slower than younger adults in the Brake condition, *t*(35.05) = −2.17, *p* = 0.037, p_fdr_ = 0.054, *d* = −0.67, 95% CI [−146.9, −5.0], and especially in the signBrake condition, *t*(34.02) = −4.44, *p* < 0.001, p_fdr_ < 0.001, *d* = −1.37, 95% CI [−248.8, −92.6]. The Brake-condition contrast did not survive FDR correction across the three post-hoc tests, whereas the signBrake contrast remained significant after correction. To isolate interference from general slowing, each participant’s braking interference cost was computed as signBrake minus Brake. This cost was more than four times larger (30 vs. 125 ms) in older adults for RT (*t*(29.54) = −7.66, *p* < 0.0001, *d* = −2.37, 95% CI [−3.15, −1.60]) and more than five times larger (1.8% vs. 9.8%) for errors (*t*(22.89) = −6.03, *p* < 0.0001, *d* = −1.89, 95% CI [−2.61, −1.18]).

### 3.2 Steering behavior

Steering-wheel angle was analyzed with a 2 (Age group: younger, older) × 2 (Time: pre-stimulus, post-stimulus) × 3 (Condition: Sign, Brake, signBrake) mixed ANOVA. Across conditions and time points (see Figure 3), older adults showed a higher steering-wheel angle than younger adults (younger: *M* = 3.57; older: *M* = 3.89), yielding a significant main effect of Age group, *F*(1,42) = 5.21, *p* = 0.028, p_GG_ = 0.028, η^2^_p_ = 0.11. No interactions involving the Age group reached significance. In each of the six Time × Condition cells, older adults tended to exhibit a higher steering-wheel angle than younger adults, with *t*-statistics between 2.09 and 2.41, *p* ≤ 0.043, p_fdr_ ≤ 0.046, and effect sizes *d* between 0.63 and 0.73. There was a strong main effect of Time, *F*(1,42) = 46.71, *p* < 0.001, p_GG_ < 0.001, η^2^_p_ = 0.53, with steering-wheel angle decreasing from pre- to post-stimulus (pre: *M* = 3.80; post: *M* = 3.66, collapsed across groups and conditions). Condition also had a significant effect, *F*(2,84) = 13.20, *p* < 0.001, p_GG_ < 0.001, η^2^_p_ = 0.24, which was further qualified by a pronounced Time × Condition interaction, *F*(2,84) = 50.95, *p* < 0.001, p_GG_ < 0.001, η^2^_p_ = 0.55. Before stimulus onset, steering-wheel angle was highest in the signBrake condition and slightly lower in the Sign and Brake conditions for both age groups. After stimulus onset, steering-wheel angle decreased overall and particularly in signBrake (younger: *M* = 3.46; older: *M* = 3.74), such that signBrake shifted from showing the highest pre-stimulus angle to the lowest post-stimulus angle relative to Sign and Brake. No higher-order interaction with Age group was significant.

**Figure 3:**
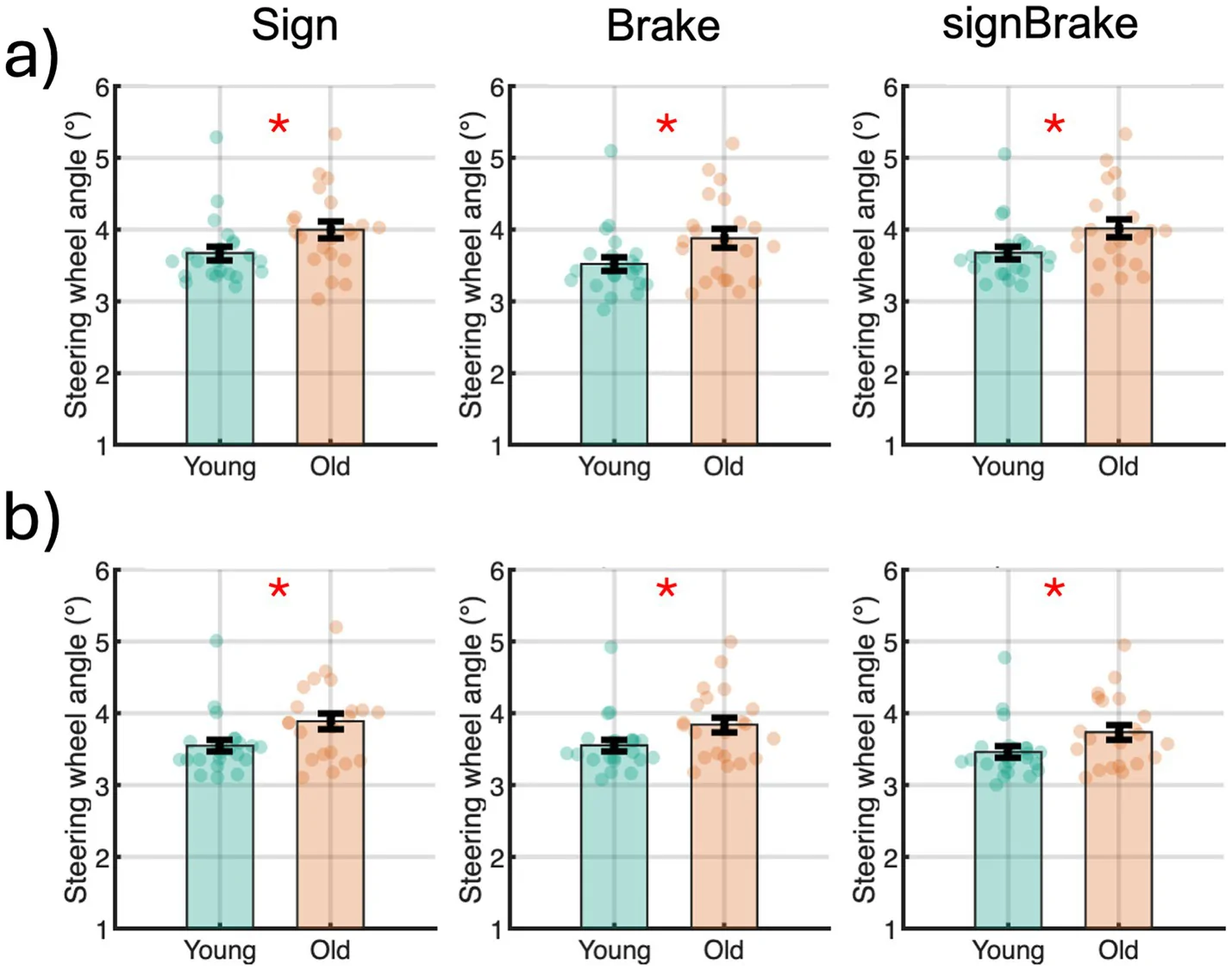
Mean absolute steering-wheel angle (degrees) as a function of age group, condition, and time. Panel **(a)** shows pre-stimulus values, and Panel **(b)** shows post-stimulus values. Bars depict group means; dots indicate individual participants, and error bars correspond to the standard error of the mean (SEM). Each subplot reports *p*-values from Welch’s *t*-test comparing age groups in each time × condition cell (* *p*fdr < 0.05). Across all cells, older adults displayed a larger steering-wheel angle than younger adults, and steering-wheel angle decreased from pre- to post-stimulus, with the largest reduction in the signBrake condition.

### 3.3 Eye-blink metrics

Eye-blink measures were averaged across the Brake, Sign, and signBrake conditions and compared between younger and older adults using Welch’s *t*-test (see Figure 4). Blink rate was higher in older than in younger adults (younger, *M* = 14.91; older, *M* = 20.22 blinks/min), but this difference was not significant, *t*(38.68) = −1.44, *p* = 0.157, *d* = −0.44, 95% CI [−12.75, 2.14]. In contrast, several temporal and amplitude-velocity parameters showed pronounced age differences. Younger adults had longer blink durations than older adults (younger, *M* = 0.279 s; older, *M* = 0.227 s), *t*(40.23) = 4.02, *p* < 0.001, *d* = 1.22, 95% CI [0.026, 0.078], as well as larger nAVR (7.41 vs. 5.99), *t*(41.98) = 3.38, *p* = 0.002, *d* = 1.02, 95% CI [0.57, 2.26], and pAVR (4.64 vs. 3.94), *t*(41.34) = 3.77, *p* < 0.001, *d* = 1.14, 95% CI [0.33, 1.08]. Closing and reopening times were also longer in younger than in older adults (closing: 0.125 vs. 0.103 s, *t*(38.02) = 3.34, *p* = 0.002, *d* = 1.02, 95% CI [0.009, 0.035]; reopening: 0.154 vs. 0.123 s, *t*(41.26) = 3.84, *p* < 0.001, *d* = 1.16, 95% CI [0.015, 0.047]). Overall, younger adults produced longer, kinematically more pronounced blinks, whereas older adults tended to blink somewhat more frequently.

**Figure 4:**
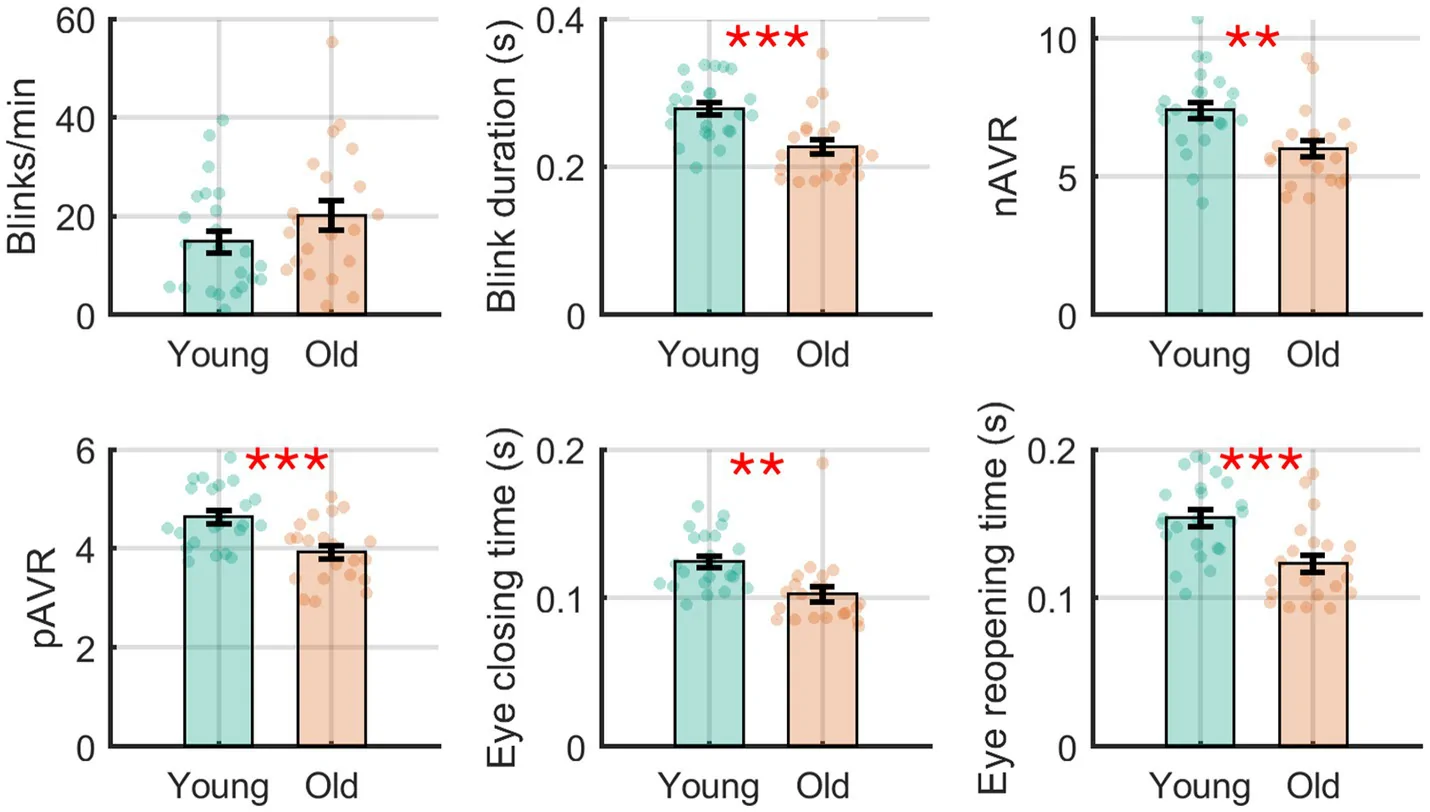
Eye-blink parameters averaged across all three task conditions (Brake, Sign, signBrake) for younger and older adults. Panels display blink rate (blinks/min), mean blink duration, negative and positive amplitude-velocity ratios (nAVR, pAVR), and eye-closing and eye-reopening times. Bars represent group means; dots show individual participants, and error bars indicate the standard error of the mean. Asterisks mark significant age differences (** *p* < 0.01, *** *p* < 0.001). Blink rate did not differ reliably between age groups, whereas younger adults showed longer and kinematically more pronounced blinks than older adults. Blink duration and eye-closing/reopening times are in s. Amplitude-velocity ratios are in centiseconds (Kleifges et al., 2017).

### 3.4 Event-related potentials

Event-related potentials (ERPs) were computed at occipital (N1), parietal (P3), and frontal (LFN) regions of interest and analyzed using mixed 2 (Age group: younger, older) × 3 (Condition: Sign, Brake, signBrake) ANOVAs on mean amplitudes (see Figure 5). The occipital N1 showed strong effects of both Condition and Age. N1 amplitudes differed markedly between conditions, *F*(2,84) = 61.16, *p* < 0.001, p_GG_ < 0.001, η^2^_p_ = 0.59, and were consistently larger in older than in younger adults across conditions, *F*(1,42) = 18.66, *p* < 0.001, p_GG_ < 0.001, η^2^_p_ = 0.31. Post-hoc comparisons indicated that N1 amplitudes were significantly smaller in the Brake than in both Sign and signBrake conditions (*p* < 0.001), whereas Sign and signBrake did not differ significantly (*p* > 0.15). The Age × Condition interaction did not reach significance, *F*(2,84) = 1.00, *p* = 0.37, p_GG_ = 0.34, η^2^_p_ = 0.02, indicating no detectable age difference in the pattern of condition-related N1 modulation. The parietal P3 amplitudes likewise varied as a function of Condition, *F*(2,84) = 7.95, *p* < 0.001, p_GG_ = 0.002, η^2^_p_ = 0.16. The Age × Condition interaction was not significant, *F*(2,84) = 1.29, *p* = 0.28, p_GG_ = 0.28, η^2^_p_ = 0.03, but the Age main effect was clearly significant, *F*(1,42) = 9.94, *p* = 0.003, p_GG_ = 0.003, η^2^_p_ = 0.19, indicating generally larger parietal P3 amplitudes in younger than in older adults. Post-hoc comparisons showed that P3 amplitudes were significantly larger for Brake than for both Sign and signBrake, whereas Sign and signBrake did not differ significantly. For the frontal LFN, there was a very strong main effect of Condition, *F*(2,84) = 20.20, *p* < 0.001, p_GG_ < 0.001, η^2^_p_ = 0.32, with the Sign condition eliciting the largest negativity (*p* < 0.001) and both Brake and signBrake showing clearly reduced negativities that did not differ from one another (*p* = 0.99). The Age main effect did not reach significance, *F*(1,42) = 2.91, *p* = 0.095, p_GG_ = 0.095, η^2^_p_ = 0.06, but the Age × Condition interaction was significant, *F*(2,84) = 3.83, *p* = 0.026, η^2^_p_ = 0.08, and remained significant under Greenhouse–Geisser correction, *F*(1.60, 67.08) = 3.83, p_GG_ = 0.035, ε = 0.80. It was driven by pronounced condition-related LFN differences in younger adults (Sign more negative than both Brake and signBrake; both *p* < 0.001) that were only trend level in older adults (*p* ≥ 0.07).

**Figure 5:**
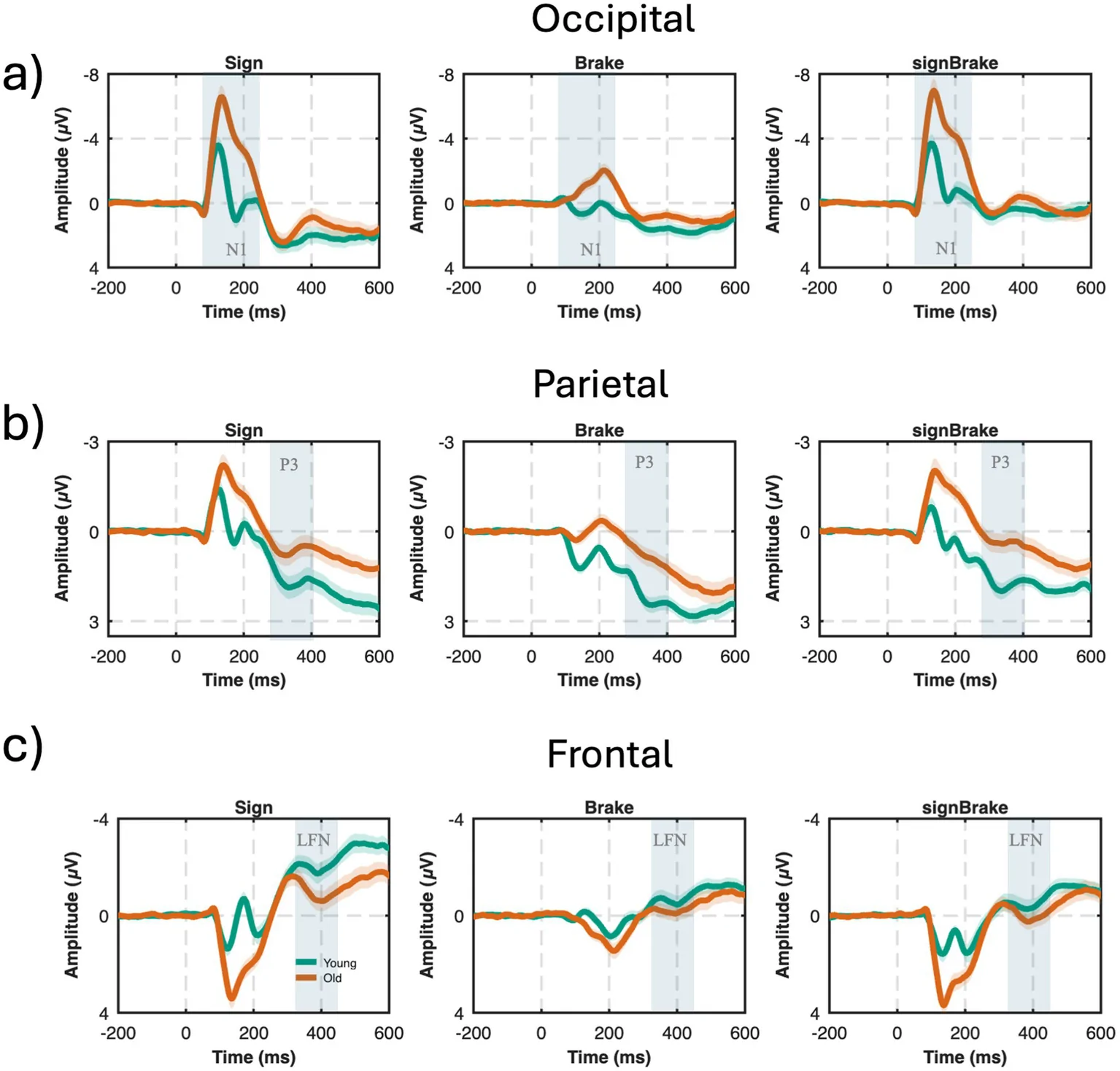
Grand-average ERPs by region, condition, and age group. Panels **(a–c)** show occipital N1 (averaged over Oz, O1, O2, PO3, POz, and PO4), parietal P3 component (averaged over P3, Pz, P4), and frontal LFN (averaged over F3, Fz, F4, FC3, FCz, and FC4) waveforms for younger (green) and older adults (orange) in the Sign, Brake, and signBrake conditions. Solid lines depict regional mean ERPs, and shaded bands indicate SEM. Gray rectangles mark the measurement windows for N1, P3, and LFN. Amplitudes are in μV and time in ms relative to stimulus onset. Waveforms are baseline-corrected to the −200 to 0 ms interval.

### 3.5 Event-related spectral perturbation

Stimulus-locked time-frequency representations of ERSP power (4–30 Hz, −400 to 1,400 ms) were averaged across all scalp electrodes and compared between younger and older drivers (see Figure 6a). For each condition (Sign, Brake, signBrake), the young-minus-old maps contained a single widespread positive cluster in the post-stimulus range, identified with a two-sided cluster-based permutation test (pixel-wise alpha = 0.025, 20,000 permutations). These clusters extended from low to higher frequencies above 4 Hz and typically covered approximately 200 to 1,200 ms after stimulus onset, indicating consistently stronger post-stimulus oscillatory power in younger than in older adults across all three tasks.

**Figure 6:**
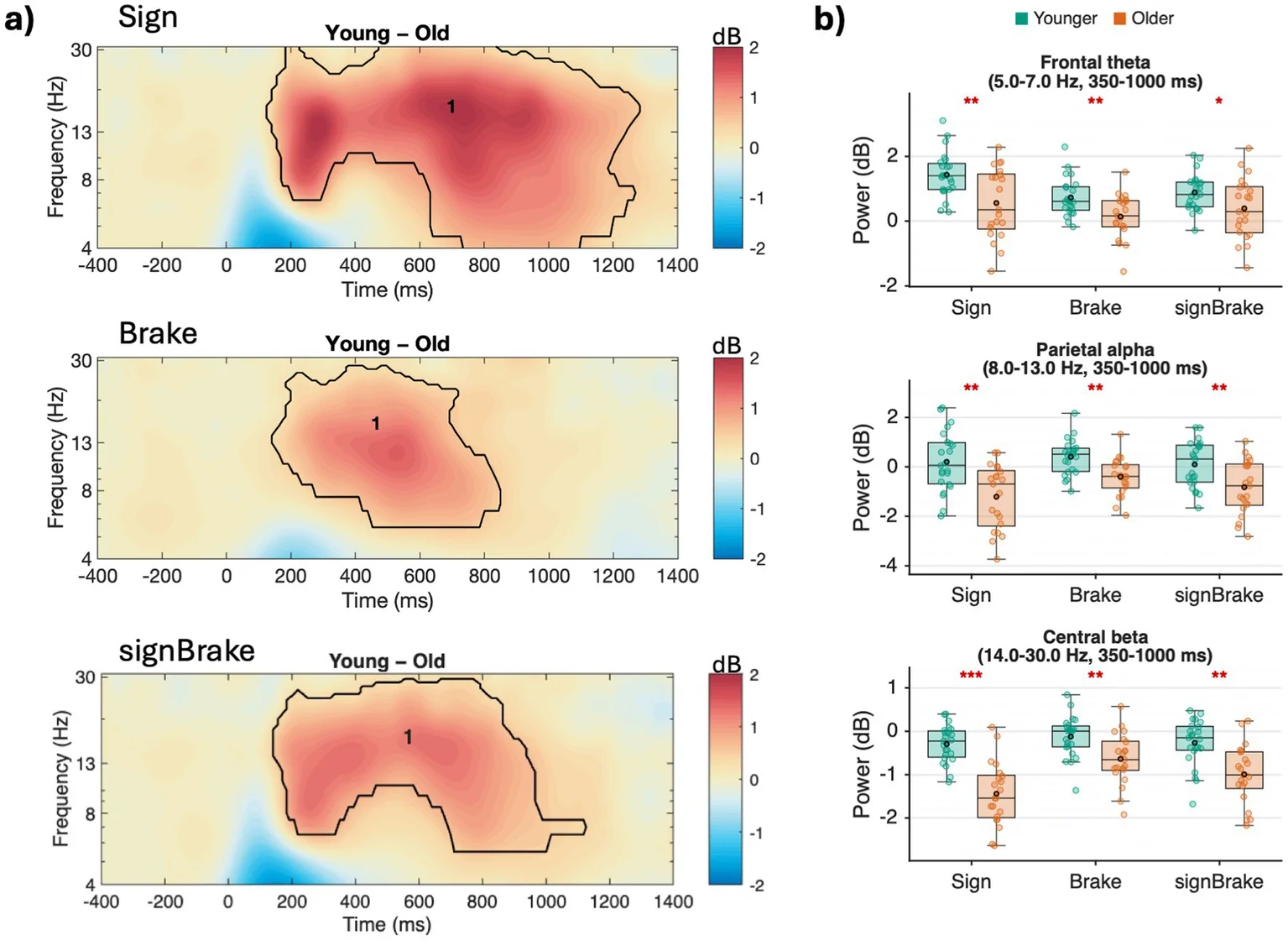
Time-frequency power differences between younger and older adults. Time-frequency representations (ERSP, dB) show younger minus older power, averaged across all scalp channels. **(a)** Displays age differences separately for the Sign, Brake, and signBrake conditions. Warm colors indicate higher power in younger than in older adults, cool colors indicate the opposite, and black contours (with cluster numbers) mark regions that reached significance in the non-parametric cluster-based permutation test (two-sided, alpha = 0.025, 20,000 permutations). **(b)** Shows region-of-interest boxplots for frontal theta (5–7 Hz), parietal alpha (8–13 Hz), and central beta (14–30 Hz) power, computed for each participant and condition as the mean ERSP within a 350–1,000 ms window. Green and orange boxplots represent younger and older adults, respectively, with dots indicating single-subject values, and black circles represent group means. Red asterisks denote significant age-group differences from Welch’s *t*-tests (two-sided, alpha = 0.05) conducted separately for each condition and frequency band (*p < 0.05, **p < 0.01, ***p < 0.001).

To quantify these effects, mean ERSP power was extracted from predefined ROIs in the 350–1,000 ms window for frontal theta (5–7 Hz), parietal alpha (8–13 Hz), and central beta (14–30 Hz; Figure 6b). Frontal theta showed significant main effects of Age (*F*(1,42) = 10.05, *p* = 0.003, p_GG_ = 0.003, η^2^_p_ = 0.19) and Condition (*F*(2,84) = 17.36, *p* < 0.001, p_GG_ < 0.001, η^2^_p_ = 0.29), but no Age × Condition interaction (*F*(2,84) = 2.12, *p* = 0.127, p_GG_ = 0.133, η^2^_p_ = 0.05). Across groups, frontal theta was highest in Sign, intermediate in signBrake, and lowest in Brake (all pairwise *p* < 0.05). Welch’s *t*-tests further showed higher frontal theta in younger than in older adults in all three conditions (all FDR corrected *p* < 0.05; *d* ≥ 0.64). Parietal alpha also showed significant main effects of Age (*F*(1,42) = 13.74, *p* < 0.001, p_GG_ < 0.001, η^2^_p_ = 0.25) and Condition (*F*(2,84) = 11.39, *p* < 0.001, p_GG_ < 0.001, η^2^_p_ = 0.21), along with a modest Age × Condition interaction (*F*(2,84) = 4.31, *p* = 0.016, p_GG_ = 0.029, η^2^_p_ = 0.09). Across both groups, parietal alpha was lower in Sign than in Brake (*p* < 0.01) and lower in signBrake than in Brake (*p* < 0.001), but Sign and signBrake did not significantly differ (*p* = 0.31). This interaction came mainly from older adults, where parietal alpha in Brake exceeded both Sign (*p* < 0.01) and signBrake (*p* < 0.05), and signBrake also exceeded Sign (*p* < 0.05). In younger adults, parietal alpha was higher in Brake than in signBrake (*p* = 0.034), but no other condition contrasts were significant (all *p* ≥ 0.55). Welch’s *t*-tests for each condition showed higher parietal alpha in younger than in older adults for all conditions (*p* < 0.01, FDR corrected; *d* ≥ 0.9).

Central beta showed the strongest combined modulation by age and condition. The ANOVA showed a main effect of Age (*F*(1,42) = 25.17, *p* < 0.001, p_GG_ < 0.001, η^2^_p_ = 0.37), a main effect of Condition (*F*(2,84) = 30.19, *p* < 0.001, p_GG_ < 0.001, η^2^_p_ = 0.42), and an Age × Condition interaction (*F*(2,84) = 13.05, *p* < 0.001, p_GG_ < 0.001, η^2^_p_ = 0.24). Across groups, central beta was lowest in Sign, fell in between in signBrake, and was highest in Brake (all *p* ≤ 0.001). This pattern came mainly from older adults, who showed highest central beta in Brake, lower in signBrake, and lowest in Sign (all *p* < 0.001). In younger adults, there were no reliable condition differences (all *p* ≥ 0.09). Welch’s *t*-tests also showed higher central beta in younger than in older adults for Sign (*p* < 0.001, FDR corrected; *d* = 1.9), Brake (*p* < 0.01, FDR corrected; *d* = 0.97), and signBrake (*p* < 0.01, FDR corrected; *d* = 1.2).

### 3.6 Multivariate age decoding

Age-related separability in the time-frequency domain was further quantified using time-resolved age-classification accuracy for each task condition and for all within- and cross-condition pairings (Figure 7). Within-condition decoding (diagonal panels, Figure 7) remained close to chance before stimulus onset, then rose clearly above 0.5 during the post-stimulus interval in all three tasks. Significant clusters were confined to the post-stimulus window in all three conditions. Corresponding weight topographies indicated that informative time-frequency patterns were distributed predominantly over frontocentral scalp regions, with some extension toward centro-parietal electrodes. Cross-condition decoding (off-diagonal panels, Figure 7) likewise yielded above-chance age classification in all six train-test pairings, indicating that the time-frequency features that distinguish younger from older adults are shared across task contexts rather than tied to any single condition. All nine within- and cross-condition pairings reached a significant post-stimulus cluster (cluster-corrected *p* < 0.05), with no significant decoding before stimulus onset in any pairing. Peak accuracy reached 0.796–0.955 within condition and 0.773–0.932 across conditions, with significant windows falling between 63 and 1,059 ms post-stimulus. To determine whether decoding strength was linked to behavioral interference, Pearson correlations were computed between each participant’s mean within-cluster classification accuracy and the braking interference cost (signBrake minus Brake RT). None reached significance in either age group or task condition (Sign: *r* = −0.09, *p* = 0.67 younger, *r* = −0.15, *p* = 0.52 older; Brake: r = 0.32, *p* = 0.14 younger, *r* = −0.17, *p* = 0.46 older; signBrake: *r* = −0.01, *p* = 0.96 younger, *r* = −0.01, *p* = 0.97 older). Given this null result, individual variation in interference cost does not appear closely tied to distributed age-related neural differences, although the limited sample size constrains sensitivity to smaller effects.

**Figure 7:**
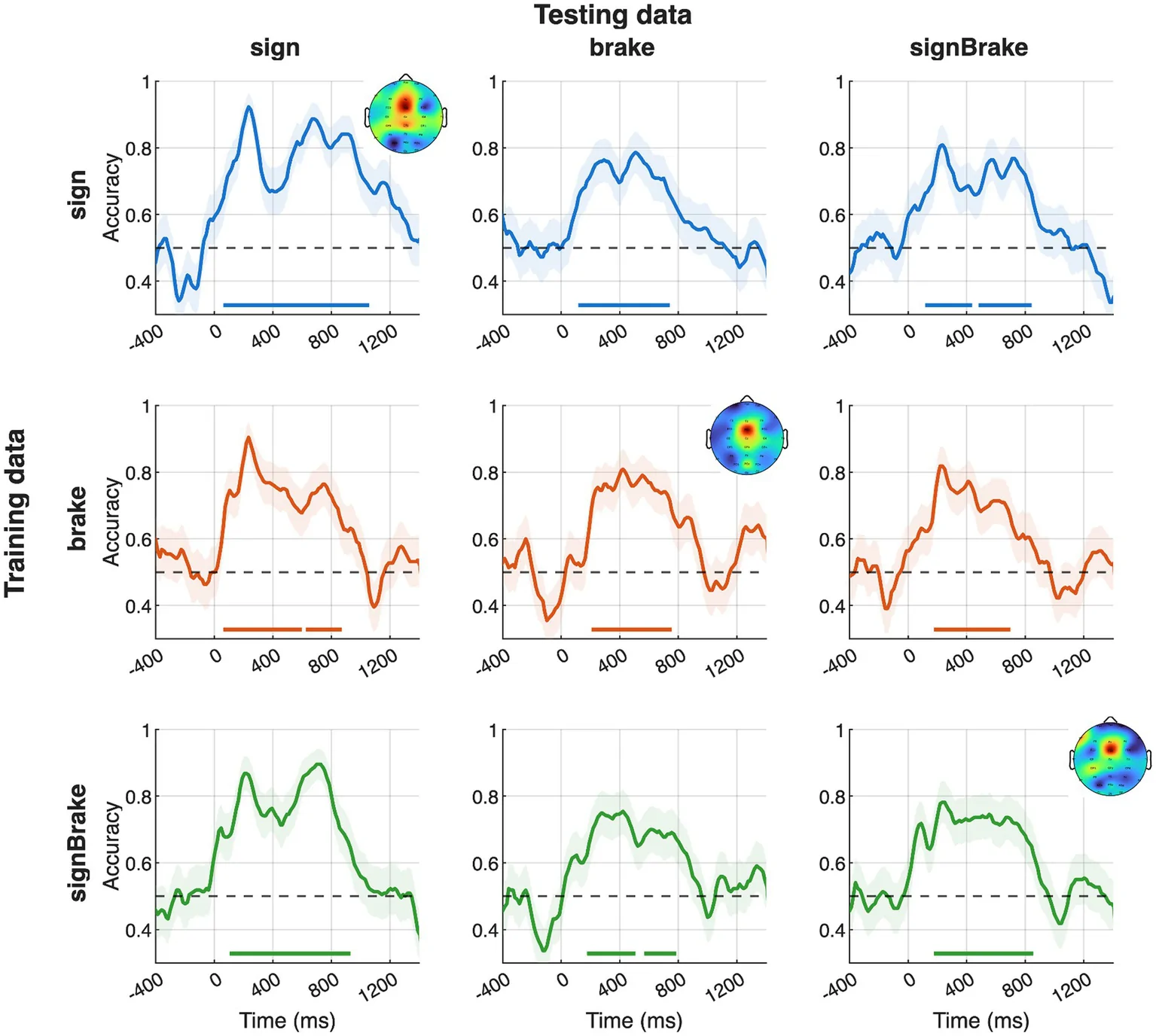
Multivariate age decoding from time-frequency power across conditions. Time-resolved classification accuracy for decoding age groups (younger vs. older adults) from broadband time-frequency power (4–30 Hz, −600 to 1,500 ms) in the three experimental conditions. Rows indicate the training condition (Sign, Brake, signBrake) while columns indicate the testing condition. Solid lines show mean decoding accuracy across participants, and shaded bands represent SEM across leave-one-subject-out decoding estimates. Horizontal colored bars at the bottom of each panel mark time intervals in which accuracy exceeded chance (0.5) according to a one-dimensional cluster-mass sign-flip permutation test across subjects (two-sided, cluster-corrected *p* < 0.05). Scalp maps in the diagonal panels depict the spatial distribution of mean absolute ridge weights, averaged across all significant time bins in the corresponding within-condition analysis, and thus highlight channels that contributed most strongly to age decoding.

## 4 Discussion

Aging selectively impaired braking prioritization under interference, and the pattern reflects reduced flexible control rather than global slowing. Older adults showed marked costs on signBrake, with higher error rates and slower brake responses, yet they did not differ from younger adults on Sign reaction times and showed very low error rates on Brake. This selective vulnerability aligns with the task rule that braking must dominate and the sign-related button press must be withheld, so the age effect most plausibly reflects reduced selective inhibition in the dual-demand context (Karthaus et al., 2020). If this age effect instead reflected a general, proportional slowing of processing speed (Salthouse, 1996), RT should slow by a similar proportion across all three conditions. Instead, it increased by 5% in Sign (889 to 934 ms, ns), 13% in Brake (575 to 651 ms), and 28% in signBrake (605 to 776 ms), the largest of the three. Using that 28% rate as a generous best-case test of proportional slowing, the interference cost would rise from only 30 ms to about 38 ms in older adults. The actual cost rose to 125 ms, more than three times that (Section 3.1). This gap points to a selective failure of interference control rather than a general reduction in processing speed. The electrophysiology supports this account, since the LFN differentiated Sign from Brake and signBrake in younger adults but showed weaker condition separation in older adults, consistent with less adaptive preparatory control settings across trial types (Kononowicz and Penney, 2016; Mäki-Marttunen et al., 2019). Taken together, the behavioral selectivity and the reduced LFN differentiation suggest that older adults prepared for the signBrake context less effectively once the stimulus arrived. That increased the reactive control demands at the moment the brake signal and sign appeared simultaneously.

The steering data add an important constraint on this control account. Older adults produced a larger steering-wheel angle across conditions, which indicates higher corrective effort during lane keeping, yet both age groups reduced their mean absolute steering-wheel angle from the pre-stimulus to the post-stimulus window, with the largest reduction in signBrake. This time-locked decrease is in line with a suppression of ongoing lateral control when a discrete event response is required, although the present data cannot distinguish this account from a biomechanical response artifact or a reduced correction rate. Drivers attenuated their continuous steering corrections when they needed to respond to the brake light or sign, and this attenuation was greatest when both responses were demanded simultaneously. Neither group showed a condition-by-age interaction in steering, so there was no evidence of a qualitatively different strategic tradeoff, and the basic priority shift from lane-keeping to braking did not differ detectably with age, consistent with earlier findings (Karthaus et al., 2018). The age effect was therefore not in the shift of motor priority toward braking, but in the precise choice of the braking action once competing sign information was present, in line with the reduced LFN condition-differentiation in the older group.

Post-stimulus oscillatory dynamics further support a model in which aging constrains control recruitment after stimulus onset. Stimulus-locked ERSP maps (Makeig et al., 2004) showed a single widespread positive cluster per condition in which younger adults exceeded older adults. Across conditions, younger adults showed stronger broadband (4–30 Hz) post-stimulus power over an extended interval, and region-of-interest analyses in the 350–1,000 ms window confirmed lower frontal theta, parietal alpha, and central beta power in older adults. Frontal theta was highest in Sign, intermediate in signBrake, and lowest in Brake in both groups. This ordering fits the role of frontal theta as a post-stimulus marker of cognitive control demand that scales with task complexity (Cavanagh and Frank, 2014), with theta tracking post-stimulus evaluation and response selection for sign information that remained present but deprioritized in signBrake. Parietal alpha was lower in Sign and signBrake than in Brake, with condition differences driven mainly by older adults. In younger adults, only the Brake vs. signBrake contrast reached significance. This fits the known role of alpha in gating attended representations and suppressing irrelevant input. Lower alpha on sign-containing trials is consistent with stronger visual engagement when a competing sign required processing or active suppression (ElShafei et al., 2020; Klimesch, 2012). Central beta showed the clearest age-by-condition modulation. Across groups, beta was lowest in Sign, intermediate in signBrake, and highest in Brake, and this gradient was carried mainly by older adults. Younger adults showed no reliable condition differences. Because beta oscillations have been linked to maintenance of the current sensorimotor set (Engel and Fries, 2010; Kilavik et al., 2013), the older-adult pattern suggests stronger tuning toward simple braking than toward interference-laden action selection, but this tuning did not prevent poorer signBrake performance. These oscillatory findings, together with the behavioral results, indicate weaker post-stimulus control recruitment in older adults and a sensorimotor set that favored simple braking over selective action under interference.

ERP results refine this interpretation by separating early sensory selection from later evaluative processing. Occipital N1 amplitudes were higher for Sign and signBrake than for Brake, which could indicate stronger early visual processing on trials that contained sign information, including the dual-demand condition. N1 was also larger in older adults across all conditions. Larger N1 amplitudes have been linked to stronger early attentional gain, so the age difference points to heightened sensory amplification at the input stage (Hillyard and Anllo-Vento, 1998; Wascher et al., 2022). Parietal P3 amplitudes were smaller in older adults across conditions, and P3 was largest for Brake relative to Sign and signBrake. Reduced P3 in older adults reflects diminished allocation to stimulus evaluation and context updating, processes that are most taxed when a rapid action decision must override competing stimulus tendencies (Polich, 1997). These two ERP age effects run in opposite directions with larger early selection and smaller late evaluation. Together they describe a pattern in which older adults do not lack sensory access to the stimulus but have reduced capacity to resolve it into a definite, context-appropriate action. The LFN adds a preparatory control-differentiation dimension, since younger adults showed clearer preparatory separation between Sign and the braking conditions whereas older adults showed weaker differentiation. N1, P3, and LFN together point to reduced preparatory flexibility and diminished evaluative processing with age. These changes converge where signBrake performance costs are largest.

Blink kinematics differed by age even though overall blink rate did not. Younger adults had longer blink durations, larger amplitude-velocity ratios, and longer opening and closing intervals. These kinematic differences reflect age-related changes in oculomotor dynamics and eyelid neuromuscular function rather than differences in blink frequency (Kleifges et al., 2017). Blink amplitude and velocity shape the temporal profile of visual suppression during each blink, so age differences in these parameters may alter how blinks punctuate attentional sampling during continuous driving tasks (Alyan et al., 2025; Caffier et al., 2003). These kinematic variations should be accounted for when using blink-derived metrics as attentional indicators in older driver research, since age-appropriate norms may be required to interpret such measures accurately.

MVPA results complement the univariate findings by showing that age-related neural differences are expressed not just in individual components or frequency bands but in the multivariate spatial distribution of post-stimulus activity. Age group decoding from time-frequency features remained near chance in the pre-stimulus period and rose reliably above chance after stimulus onset across all tasks, with the broadest intervals and highest peak accuracies in the post-stimulus window. Weight topographies indicated a predominance of frontocentral contributions extending toward centro-parietal sites, which aligns with the P3 age effect and the widespread post-stimulus power differences. Cross-condition decoding likewise succeeded across multiple train-test pairings rather than being tied to a single condition, indicating that the distributed age-related signature is carried across task contexts. These patterns are therefore best read as evidence for a recurring distributed structure rather than as precise estimates of accuracy or of cross-condition transfer length. Age decoding rose post-stimulus even in conditions where behavioral differences were smaller, which suggests that older adults may sustain performance through altered neural patterns rather than by preserving the same cognitive operations as younger adults (Cichy and Pantazis, 2017; Wan et al., 2024).

The pattern has direct implications for driver safety. The selective rise in signBrake-related errors and braking delays in older adults points to a vulnerability in the type of situation that most closely reflects real-world driving interference, where a safety-critical braking signal competes with concurrent visual information. As described above, the time-locked reduction in mean absolute steering-wheel angle at event onset showed no detectable age interaction, so no evidence emerged that the vehicle-level tendency to suppress ongoing lateral control in favor of a discrete braking response changes with age. The risk is not that older adults fail to prioritize driving, but that they have greater difficulty selecting the correct action when interference is present (Carr and Babulal, 2023; Doroudgar et al., 2017). This profile suggests that interventions are most effective when they support early selection and inhibition around interference-laden events, rather than focusing primarily on general response slowing or lane-keeping performance.

These results fit within a broader picture of behavioral and physical risk factors in older drivers. Older drivers commonly self-regulate by avoiding higher-demand situations, such as night driving, congested traffic, and unfamiliar routes, and by limiting in-vehicle distractions; these adaptations partly offset age-related declines in attention and control (Carr and Babulal, 2023). Elevated crash and injury rates per mile in this group reflect not only more frequent errors but also greater physical vulnerability, so that a given collision is more likely to cause serious injury than in a younger driver (Tefft, 2017). The present data add a mechanistic dimension to this picture. The situations older drivers most often avoid are precisely those that load interference-related action selection, the process most affected by aging in the present data.

Several design features limit the scope of these findings. The fixed-base simulator and controlled car-following environment offered high experimental control but lacked vestibular cues and the unpredictable hazards of real driving, which may restrict generalizability to on-road behavior. In signBrake trials, the sign and brake signals always appeared simultaneously. This fixed timing may exaggerate interference by removing any temporal priority for the brake signal. Varying the stimulus onset asynchrony in future work would clarify whether giving the brake a small temporal lead reduces the age differences observed here. MVPA identifies neural patterns that discriminate age groups but does not specify the cognitive processes generating them. Because decoding strength was unrelated to interference cost, the two measures appear dissociable at the individual level, although the modest sample constrains power. Capturing a link between them may call for trial-level rather than subject-level modeling. Within-subject and interaction effects were detectable at *f* ≥ 0.19, but between-subject age comparisons required large effects to achieve 80% power (Cohen’s *f* ≥ 0.35; *d* ≥ 0.87). Smaller between-group differences may therefore have gone undetected. A larger sample would improve sensitivity for between-group comparisons and support subgroup analyses. Future work could also extract condition-specific blink indices, link trial-level EEG and steering dynamics to braking outcomes within each condition, and test whether the age-decoding features that generalize across conditions predict within-group variance in braking errors and latencies. Stimulus density was higher than typical on-road event rates and may have shifted attention toward discrete events, possibly more so in older adults who are known to be more sensitive to interference. The signBrake age effects reported here should therefore be read as the maximal interference cost under high event density. Session order was identical across age groups, so any practice, fatigue, or session-order effects acted equivalently on both groups and cannot account for the age or Age × Condition contrasts reported here.

## 5 Conclusion

Older adults were selectively impaired when braking required priority under interference. In signBrake trials, they responded more slowly and made more errors, while age differences were smaller in single-demand conditions. On the neural side, older adults showed weaker LFN differentiation across task demands, smaller P3 amplitudes, and lower post-stimulus power in frontal theta, parietal alpha, and central beta bands, with condition-related modulation most pronounced in the older group. MVPA decoded age after stimulus onset and generalized across conditions. The age-related neural pattern held across task contexts. The findings suggest that aging constrains action selection under interference during safety-critical braking. This pattern supports a focus on early selection and inhibition in older drivers.

## Data Availability

The raw data supporting the conclusions of this article will be made available by the authors, without undue reservation.
